# Effect of QT interval-prolonging drugs taken in pregnancy on the neonatal QT interval

**DOI:** 10.3389/fphar.2023.1193317

**Published:** 2023-08-07

**Authors:** Holger Michel, Antonia Potapow, Markus-Johann Dechant, Susanne Brandstetter, Sven Wellmann, Angela Köninger, Michael Melter, Christian Apfelbacher, Michael Kabesch, Stephan Gerling, the KUNO-Kids study group

**Affiliations:** ^1^ University Children’s Hospital Regensburg (KUNO), Hospital St. Hedwig of the Order of John, University of Regensburg, Regensburg, Germany; ^2^ Member of the Research and Development Campus Regensburg (WECARE), Hospital St. Hedwig of the Order of St. John, Regensburg, Germany; ^3^ Clinic of Obstetrics and Gynecology St. Hedwig, University of Regensburg, Regensburg, Germany; ^4^ Institute of Social Medicine and Health Economics, University of Magdeburg, Magdeburg, Germany

**Keywords:** acquired long QT, neonate, KUNO-Kids, maternal medication, ECG

## Abstract

**Introduction:** Acquired QT interval prolongations due to drug side effects can result in detrimental arrhythmia. Maternal use of placenta-permeable drugs may lead to fetal exposure, thus leading to an increased risk of neonatal QT prolongation and arrhythmia.

**Objectives:** This study aimed to evaluate the influence of maternal QT-prolonging medication on the neonatal QT interval.

**Methods:** In the prospective KUNO-Kids health study, an ongoing population-based birth cohort, we classified maternal medications according to the known risk of QT interval prolongation. Effects on the neonatal QT interval were tested by linear regression analyses, correcting for perinatal confounders (birth weight, gestational age, birth mode, and age at ECG recording). Subgroup analyses were performed for selective serotonin reuptake inhibitors, proton pump inhibitors, and antihistamine dimenhydrinate. Logistic regression analysis was performed using a QTc of 450 ms as the cut-off value.

**Results:** A total of 2,550 pregnant women received a total of 3,990 medications, of which 315 were known to increase the risk of QT prolongation, resulting in 105 (4.1%) neonates exposed in the last month of pregnancy. Overall, the mean age of the neonates at ECG was 1.9 days and the mean QTc (Bazett) was 414 ms. Univariate (regression coefficient −2.62, *p* = 0.288) and multivariate (regression coefficient −3.55, *p* = 0.146) regression analyses showed no significant effect of fetal medication exposure on the neonatal QT interval, neither in the overall nor in the subgroup analysis. Logistic regression analysis showed no association of exposure to maternal medication with an increased risk of neonatal QT interval prolongation (OR (odds ratio) 0.34, *p* = 0.14).

**Conclusion:** The currently used maternal medication results in a relevant number of fetuses exposed to QT interval-prolonging drugs. In our cohort, exposure was found to have no effect on the neonatal QT interval.

## 1 Introduction

A severely prolonged QT interval is associated with life-threatening complications, such as stillbirth and sudden infant death syndrome (SIDS) in infants, and detrimental arrhythmia and sudden unexplained death (SUD) in children and adults (Schwartz et al., 1998; 2001; Arnestad et al., 2007; Tester and Ackerman, 2007; Tester et al., 2012). Approximately 10% of SIDS cases can be attributed to long QT syndrome (LQTS) (Arnestad et al., 2007), a genetic cardiac ion-channel disease with a prevalence of about 1:2000 (Schwartz et al., 2009) for which associated mutations in 17 genes have been described (Wilde et al., 2022). In addition to genetically caused LQTS, QT prolongation can also be acquired as an adverse effect of certain drugs. Through various interactions with cardiac ion channels, these drugs can prolong the QT interval and thus increase the risk for critical arrhythmia. Furthermore, genetic variants are known to influence individual susceptibility to drug-induced QT interval prolongation (Kallergis et al., 2012).

QT interval prolongation has been described for several drugs commonly taken by pregnant women, including selective serotonin reuptake inhibitors (SSRIs) (Funk and Bostwick, 2013), proton pump inhibitors (PPIs) (Moore et al., 1989; Gau et al., 2012; Bibawy et al., 2013), and antihistamine dimenhydrinate (diphenhydramine) (Poluzzi et al., 2015). Maternal drug use during pregnancy may result in fetal exposure if drugs pass the placenta. In the case of pro-arrhythmogenic drugs, this may lead to an increased risk of arrhythmia in the fetus and neonate. However, data on the influence of potential QT interval-prolonging maternal medication on the postnatal neonatal QT interval are very limited. There are some case reports on neonates with markedly prolonged postnatal QT intervals that were possibly related to exposure to maternal medication. One of these patients needed therapy for life-threatening arrhythmia and torsade de pointes, occurring after maternal intake of tricyclic antidepressants (Dubnov, 2005; Fukushima et al., 2016; Leerssen et al., 2019). For SSRIs, two case–control studies addressed the issue of neonatal QT interval prolongation after intrauterine exposure with inflicting results (Dubnov-Raz et al., 2008; Lindsay-Sutherland, 2021).

Apart from a possible influence of maternal medication on the neonatal electrocardiogram (ECG), there are other neonatal and maternal factors that may affect the neonatal QT interval. For example, studies have reported a variable effect of gestational age on neonatal repolarization (Marcellino et al., 2021; Pærregaard et al., 2021), whereas maternal age at delivery had no impact (Pærregaard et al., 2022). The interpretation in newborns is methodologically challenging as the QT interval varies with age and may change within days (Schwartz et al., 1982; Simma et al., 2020). Therefore, the aim of this study was to evaluate the influence of peripartum maternal QT interval-prolonging medication on the QT interval in the neonatal ECG, taking peripartum confounders into account.

## 2 Materials and methods

### 2.1 Study design

The KUNO-Kids health study is a population-based prospective birth cohort study carried out at the Hospital St. Hedwig of the Order of John, Regensburg, Germany, as described elsewhere (Brandstetter et al., 2019). In the presented study, we analyzed parental and neonatal demographics, maternal medication, and postnatal ECGs recorded from 3,181 participants. Written informed consent was obtained for each case. The study was approved by the Ethics Committee of the University of Regensburg (14-101-0347).

### 2.2 Study population

All neonates born between the beginning of the KUNO-Kids health study, 27 July 2015, and 31 December 2019 were eligible. We included neonates with a postnatal ECG and a complete documentation of the maternal medication during pregnancy. Exclusion criteria were as follows: outpatient childbirth, stillbirth, maternal age less than 18 years, or maternal German language skills inadequate to achieve informed consent.

### 2.3 Exposure to maternal medication and perinatal demographics

Maternal medication during pregnancy was recorded in an interview conducted postnatally by the study team and verified by checking the maternity pass. For each drug, the month of gestation and the frequency of intake were recorded. Based on the manufacturer’s information and classification on the CredibleMeds^®^ website (RL Woosley et al., 2022.), drugs were classified as having or not having a known risk for QT interval prolongation. Participants with maternal drugs that could not be classified due to insufficient specifications were excluded from the analysis.

Infants were defined as exposed (Med+) if a medication with a known risk of QT interval prolongation was taken by the mother at least once daily during the last month of pregnancy; otherwise, they were assigned to the unexposed (Med-) group. Among drugs with the known risk of QT interval prolongation, a subgroup analysis was performed for SSRIs, PPIs, and dimenhydrinate only.

Data on maternal demographics (age and migration background), neonatal characteristics (gestational age, birth weight, and sex), and the mode of birth (spontaneous delivery and caesarian section) were collected from patient records and a postpartum interview.

### 2.4 ECG

ECGs were recorded in the first week of life, as described by Simma et al. (2020), according to standard operating procedures. The 12 lead ECGs were performed with a commercially available recording device (MAC 5500 HD^®^, GE Healthcare, Freiburg, Germany), 10 adhesive electrodes (Ambu^®^ BlueSensor NF50-A/12, Ambu, Bad Nauheim, Germany), and recorded at a paper with a speed of 50 mm/s including an additional rhythm recording at 25 mm/s. All ECG records were evaluated or revised by experienced pediatric cardiologists (SG and HM). The QT interval was measured manually from the onset of the Q-wave to the end of the T-wave using the tangent method. It was corrected for time (QTc) using Bazett’s formula (QTc [ms] = QT interval [ms]/(√RR [s]/1 [s])), as recommended by the guidelines for the interpretation of neonatal ECGs of the European Society of Cardiology (Schwartz, 2002). QTc was measured in lead II, and in the case of a value >440 ms, a mean of lead II, V5, and V6 was calculated according to Schwartz et al. B). The child’s age at the time of the ECG recording, QRS width, and heart rate were documented.

### 2.5 Statistical analysis

Parental and neonatal demographics, perinatal characteristics, and ECG parameters were entered in an electronic case report form (eCRF), and extensive plausibility checks were performed. We tested the hypothesis that fetal exposure to maternal QT-prolonging medication is associated with a prolonged neonatal QT interval using linear regression analysis. Potentially confounding variables (birth weight, gestational age, birth mode, and age at ECG) were included using a multivariate model. In a sensitivity analysis, we tested whether maternal medication is associated with a QTc interval larger than 450 ms using logistic regression analysis. The same set of confounder variables was included. All descriptive statistics and univariate and multivariate regression analyses were computed using IBM SPSS statistics (version 28).

## 3 Results

Out of a total of 3,181 participants in the KUNO-Kids health study, 631 were excluded because of incomplete or unclassifiable documentation of maternal medication or a lack of ECG recording. Therefore 2,550 participants were included in this analysis. A total of 22 additional participants had to be excluded in the multivariate analysis due to the incomplete dataset on perinatal demographics. A total of 2,528 participants were included in the multivariate analysis.

On average, mothers were 33 years old (SD = 4.4 years, range 18–49 years). An immigration background was present in 18.2% (n = 464) of the families. The proportion of caesarean delivery was 29.1% (n = 742). The mean birth weight of the neonates was 3366 g (SD = 503 g, range 1650 g–4985 g), and the mean gestational age was 39.5 weeks (SD = 1.5 weeks, range 33.3–43.1 weeks). An estimate of 48.7% of infants were female and 51.3% were male. The mean age of the neonates at the time of ECG was 1.9 days (SD = 0.82 days, range 1–7 days). The mean heart rate was 117.6 beats per minute (SD = 17.0, range 70–195), and QRS duration was 59 ms on average (SD = 4.3 ms, range 40–110 ms). The cohort characteristics are summarized in Table 1.

**TABLE 1:** Cohort characteristics, total n = 2,550 participants.

Variable	n (%)	Mean (SD)
Maternal age		33 years (4.4)
Migration background	464 (18.2%)	
Caesarean delivery	742 (29.1%)	
Birth weight		3,366 g (503)
Male	1,308 (51.3%)	
Female	1,242 (48.7%)	
Gestational age		39.5 weeks (1.5)
Neonatal ECG		
Age at the ECG recording		1.9 days (0.82)
Heart rate		118 bpm (17)
QRS duration		59 ms (4.3)
QTc		414 ms (24)

ECG, electrocardiogram; QTc, corrected QT interval using Bazett’s formula; SD, standard deviation.

A total of 3,990 entries for maternal medication were reported and classified. In total, 315 drugs were classified as representing a known risk for QT interval prolongation. A total of 105 participants were assigned to the drug exposure group (Med+) with a daily maternal intake of a potential QT interval-prolonging drug in the last month of pregnancy. Table 2 shows a summary of the drugs assigned to the Med+ group. A total of 2,445 participants were assigned to the non-exposure group.

**TABLE 2:** Drugs assigned to the group with the known risk of QT interval prolongation and the number of participants with peripartum fetal exposure.

Medication group	Active agent	Fetal exposure, n (%)
Anticonvulsants	Levetiracetam	5 (0.2)
Beta-agonist inhalation	Reproterol, salmeterol, and fenoterol	17 (0.7)
Neuroleptics	Quetiapine	3 (0.1)
Nose drops	Xylometazoline	1 (<0.1)
Proton pump inhibitor	Pantoprazole and omeprazole	28 (1.1)
Serotonin norepinephrine reuptake inhibitor	Duloxetine, reboxetine, and venlafaxine	9 (0.4)
Selective serotonin reuptake inhibitors	Citalopram, escitalopram, fluoxetine, paroxetine, and sertraline	30 (1.2)
Tricyclic antidepressants	Amitriptyline, opipramol, clomipramine, doxepine, imipramine, nortriptyline, and trimipramine	9 (0.4)
Antiemetics	Dimenhydrinate	16 (0.6)
Antibiotics	Ofloxacin and ciprofloxacin	3 (0.1)
Macrolide antibiotics	Clarithromycin, azithromycin, and erythromycin	3 (0.1)

**FIGURE 1:**
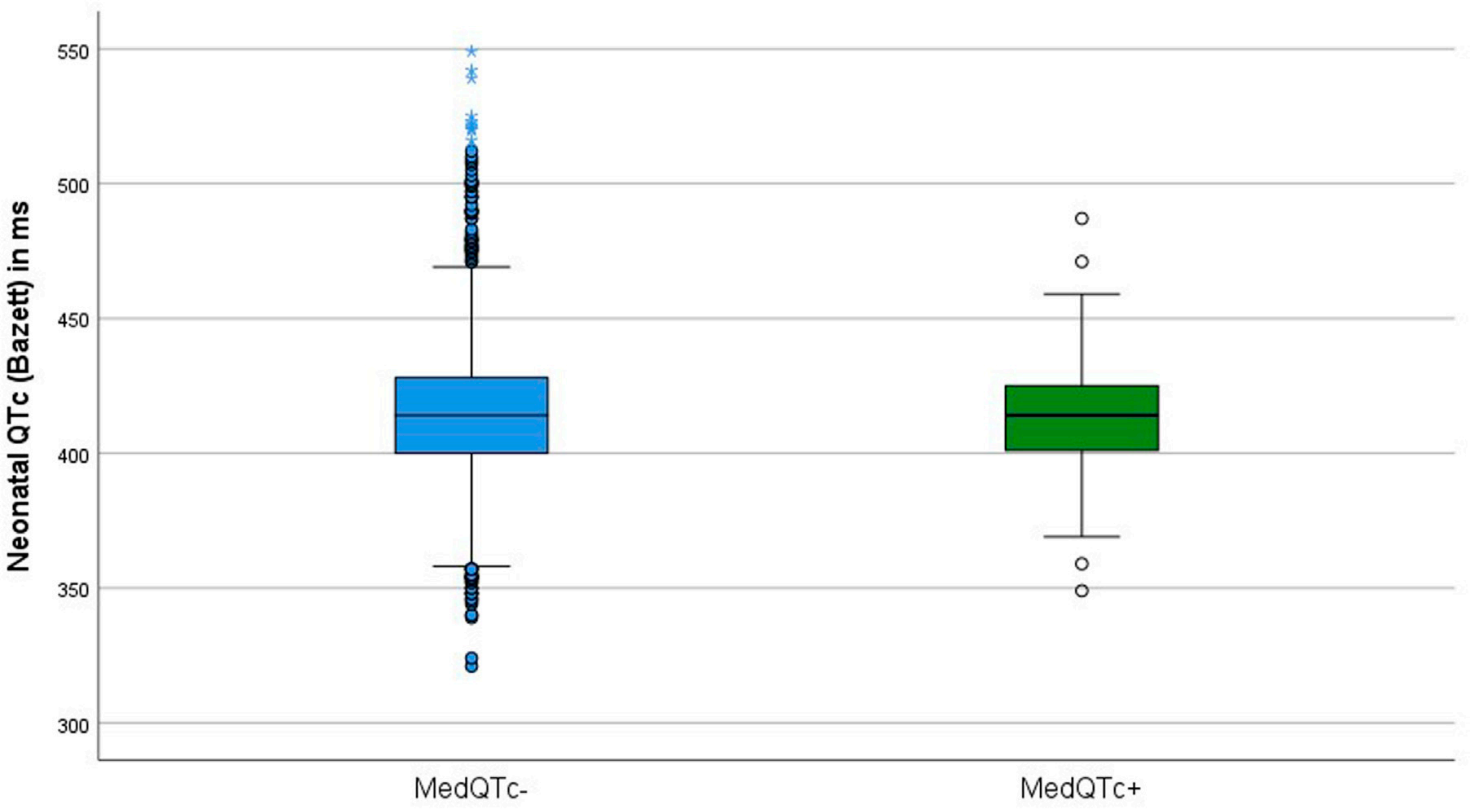
Boxplot of the neonatal QTc intervals of neonates prenatally exposed (Med+ n = 105) and unexposed (Med- n = 2,445) to QT interval-prolonging maternal medication.

Overall, mean neonatal QTc was 414 ms (SD = 24.4 ms, range 321–549 ms). The mean QTc of participants with or without exposure to maternal QT interval-prolonging medication was 412 ms (SD = 20.4 ms, range 349–487 ms) and 414 ms (SD = 24.6 ms, range 321–549 ms), respectively, (Figure 1). Using 450 ms as the cut-off, the QTc interval was prolonged in 4.6% (n = 117) of the participants (4.6%), with a rate of 4.7% (n = 114) in the Med- group and 2.9% (n = 3) in the Med+ group. Univariate analysis showed no statistically significant association between the neonatal QT interval and exposure to maternal medication (regression coefficient −2.62, *p* = 0.288). To control the possible influence of confounding variables, we performed a multivariate analysis, including birth weight, gestational age, birth mode, and age of the neonate at the time of the ECG recording. Table 3 shows that even after controlling these confounding variables, there was no significant association between exposure to maternal medication and the neonatal QT interval (regression coefficient −3.55, *p* = 0.146). Each variable considered as a confounder showed a statistically significant association with the postnatal QT interval using the multivariate model. In detail, the postnatal QT interval was decreasing with increasing gestational age (regression coefficient −2.67, *p* < 001) and with increasing age of the infants at the time of the ECG (regression coefficient −2.37, *p* < 0.001). An increase in birth weight was associated with a longer QT interval (regression coefficient 0.003, *p* = 0.003). A caesarean section was associated with a significantly longer postnatal QT interval (regression coefficient 3.03, *p* = 0.006).

**TABLE 3:** Multivariate linear regression analysis on the effect of exposure to peripartum maternal QT-prolonging drugs on the neonatal QT interval (highlighted in bold letters), correcting for perinatal confounders such as birth weight (in gram), gestational age (in weeks), the mode of delivery (caesarean section/spontaneous delivery), and age of the neonate at the ECG recording (in days). Total n = 2,528 participants.

	Unstandardized coefficient		Standardized coefficient		
	B (95% CI)	SE	Beta	t	Significance (p-value)
Constant	512 (485–539)	13.84			
**Peripartum exposure QT-prolonging drugs**	**−3,55 (-8,33–1,24)**	**2,44**	**−0,029**	**−1,45**	**0,146**
Birth weight (gram)	0.003 (0.001–0.006)	0.001	0.069	2.99	0.003
Gestational age (weeks)	−2.67 (−3.42 to −1.91)	0.384	−0.165	−6.94	<0.001
Birth mode	3.03 (0.855–5.2)	1.11	0.056	2.73	0.006
Age at ECG (days)	−2.37 (−3.58 to −1.17)	0.62	−0.079	−3.86	<0.001

Due to limitations to the sample size, subgroup analyses were performed for PPIs, SSRIs, and dimenhydrinate only, with 26, 29, and 16 exposed participants, respectively. Compared with unexposed neonates, no subgroup showed significantly prolonged QT intervals in the neonatal ECG. After exposure ro PPIs and SSRIs the mean postnatal QTc was shorter, with 408 ms (SD = 15 ms, range 369 ms–435 ms) and 408 ms (SD = 27 ms, range 349 ms–471 ms), respectively. After maternal intake of dimenhydrinate, the mean neonatal QTc interval was slightly longer at 419 ms (SD = 11.3 ms, range 398 ms–433 ms). In multivariate analysis, these differences were also not significant for any subgroup (Table 4).

**TABLE 4:** Multivariate subgroup analysis of the effect of exposure to the respective peripartum maternal drugs on the neonatal QT interval (highlighted in bold letters), correcting for perinatal confounders such as birth weight (in gram), gestational age (in weeks), the mode of delivery (caesarean section/spontaneous delivery), and age of the neonate at the ECG recording (in days).

	Unstandardized coefficient		Standardized coefficient		Significance (p-value)
	B (95% confidence interval)	Std. error	Beta	t	
Constant	514 (486–542)	14.2		36.3	<0.001
**Peripartum exposure proton pump inhibitor**	**−6.97 (-16.3–2.4)**	**4.77**	**−0.29**	**−1.46**	**0.144**
Birth weight (gram)	0.003 (0.001–0.005)	0.001	0.066	2.81	0.005
Gestational age (weeks)	−2.71 (−3.47–−1.94)	0.392	−0.166	−6.9	<0.001
Birth mode	2.96 (0.74–5.2)	1.14	0.055	2.61	0.009
Age at ECG (days)	−2.26 (-3.5–−1.02)	0.63	−0.075	−3.58	<0.001

	Unstandardized coefficient		Standardized coefficient		Significance (p-value)
	B (95% confidence interval)	Std. error	Beta	t	
Constant	513 (485–541)	14.2		36.1	<0.001
**Peripartum exposure SSRI**	**−6.86 (-15.8–2.1)**	**4.56**	**−0.3**	**−1.5**	**0.132**
Birth weight (gram)	0.003 (0.001–0.006)	0.001	0.067	2.82	0.005
Gestational age (weeks)	−2.69 (−3.46–−1.92)	0.394	−0.165	−6.8	<0.001
Birth mode	2.90 (0.66–5.14)	1.14	0.053	2.55	0.011
Age at ECG (days)	−2.29 (−3.5–−1.06)	0.63	−0.076	−3.63	<0.001

	Unstandardized coefficient		Standardized coefficient		Significance (p-value)
	B (95% confidence interval)	Std. error	Beta	t	
Constant	514 (486–542)	14.2		36.2	<0.001
**Peripartum exposure dimenhydrinate**	**3.71 (-8.2–15.6)**	**6.08**	**0.012**	**0.611**	**0.541**
Birth weight (gram)	0.003 (0.001–0.005)	0.001	0.063	2.67	0.008
Gestational age (weeks)	−2.7 (−3.47–−1.93)	0.394	−0.165	−6.85	<0.001
Birth mode	2.96 (0.72–5.19)	1.14	0.054	2.6	0.01
Age at ECG (days)	−2.21 (−3.5–−0.97)	0.64	−0.073	−3.5	<0.001

A, exposure to proton pump inhibitors (n = 26); B, exposure to selective serotonin reuptake inhibitors (n = 29); C, exposure to dimenhydrinate (n = 16)

Increased genetic susceptibility to drugs that induce QT interval prolongation in some individuals at risk could lead to an increased rate of QT prolongation without significantly affecting the QT interval of the whole group of exposed neonates. To test whether exposure to maternal QT prolonging medication is associated with an increased rate of prolonged neonatal QT interval, a logistic regression analysis was additionally performed using a QTc of 450 ms as the cut-off value. In this analysis, controlling gestational age, birth weight, birth mode, and age at ECG as confounders, exposure to maternal medication was not associated with an increased chance of neonatal QTc prolongation above 450 ms (OR (odds ratio) 0.34, 95% confidence interval 0.083–1,415, *p* = 0.14) (Table 5).

**TABLE 5:** Logistic regression analysis on the effect of exposure to peripartum maternal QT-prolonging drugs (highlighted in bold letters) on the rate of neonatal QT interval prolongation (QTc > 450 ms), correcting for perinatal confounders such as birth weight (in gram), gestational age (in weeks), the mode of delivery (caesarean section/spontaneous delivery), and the age of the neonate at the ECG recording (in days).

	Odds ratio	95% confidence interval		Significance
		Lower bound	Upper bound	(p-value)
Constant	8,168.82			<0.001
**Peripartum exposure QT-prolonging drugs**	**0.34**	**0.08**	**1.42**	**0.139**
Birth weight (gram)	1,00	1,00	1.001	0.073
Gestational age (weeks)	0.72	0.62	0.83	<0.001
Birth mode	1.65	1.103	2.45	0.015
Age at ECG (days)	0.81	0.636	1.031	0.087

## 4 Discussion

Through various pathophysiological mechanisms, drugs can lead to QT interval prolongation and subsequently to an increased risk of critical arrhythmias, mostly through interactions with cardiac ion channels. Studies in adults estimate that 5%–7% of ventricular tachycardia, ventricular fibrillation, and sudden cardiac death are due to drug-induced LQTS and torsade de pointes (Kallergis et al., 2012). Maternal drug use during pregnancy may result in fetal exposure via the placenta.

The aim of this prospective birth cohort study was to investigate the influence of the peripartum maternal use of potential QT interval-prolonging medication on the neonatal ECG. As a main finding, there was no significant effect on the mean neonatal QT interval and on the rate of infants with QT prolongation.

In our cohort, a relevant number of infants (n = 105, 4.1%) were exposed to potential QT interval-prolonging drugs by maternal intake in the last 4 weeks of pregnancy, for which placental transfer is known. Most frequent was the maternal use of SSRIs, PPIs, beta-agonist inhalation, and dimenhydrinate, some of which are common over-the-counter medications in Germany.

Despite heterogeneous exposure due to the use of a variety of different drugs in our cohort, in a first general approach, we tested the effect of all potential QT interval-prolonging drugs in a combined analysis. No significant change in the mean neonatal QT interval after exposure to maternal potential QT interval prolonging-drugs was found. The rate of neonates with a QTc interval greater than 450 ms was also not significantly higher in the exposed group than that in the unexposed neonates. This cut-off value has been recommended by Saul et al. (2014) for follow-up ECGs and further evaluation. The majority of ECGs in our study were recorded on the second and third days after birth (mean age = 1.9 days, SD = 0.82 days). Therefore, we cannot exclude that possible effects on the neonatal QT interval may already have disappeared over time due to decreasing drug levels in the neonate. In our institution, most neonates are discharged from the maternity ward on the third day of birth. Thus, our study indicates that at this time point, there was no increased risk of arrhythmia or need for further diagnostic evaluation due to drug-induced QT prolongation for the infants.

Second, since numerous additional factors may have an influence on the neonatal QT interval, we performed a multivariate analysis to correct for confounding factors. This analysis also showed no significant influence of maternal QT interval-prolonging medication on the neonatal QT interval. In contrast, there was a significant association between the confounding factors of gestational age, birth weight, birth mode, and timing of the ECG with neonatal QT intervals in our setting.

Transient postnatal QT interval prolongation and high dynamics in the first few days with normalization over the course of days have been described (Schwartz et al., 1982; Simma et al., 2020); thus not surprisingly, in this analysis, the neonatal QT interval decreased with increasing postnatal age of the neonates. An influence of gestational age on the QT interval has been studied previously, with various effects depending on the level of prematurity and the timing of the ECG recording (Séguéla et al., 2012; Marcellino et al., 2021; Pærregaard et al., 2021). The study of Marcellino et al. showed no difference in the QT interval with respect to gestational age. Pærregaard et al. demonstrated a slight prolongation of the QT interval in infants at >41 compared with <35 weeks of gestation. In contrast, Séguéla et al. found that in preterm infants, QT intervals increased up to a peak at 32 weeks of gestation and became shorter again thereafter. In our study, neonates from 33 to 43 weeks of gestation were included, and in the multivariate approach similar to the latter study, there seemed to be a decrease in the postnatal QT interval with increasing gestational age. Furthermore, in our analysis, increasing birth weight was associated with an increasing QT interval, but this effect was rather small, with an average of a 3-ms increase in the QT interval per kilogram of weight. Previous studies did not find such a difference (Séguéla et al., 2012; Pærregaard et al., 2021). One possible explanation for the effect in our data might be that we did not correct for gestational diabetes of the mother. After this complication of pregnancy, prolonged QT and QT dispersion have been described and neonates also tend to be heavier (Al-Biltagi et al., 2021). Interestingly, our analysis seemed to show a difference in the neonatal QT interval with respect to the mode of delivery, spontaneous delivery vs. cesarean section, although the observed effect was small (increase in QTc by 3.03 ms in infants after cesarean section vs. spontaneous delivery). In the logistic regression analysis, the rate of neonates with a QTc interval above 450 ms was also significantly increased in children born by cesarean section compared to spontaneous delivery (odds ratio 1.65; Table 5). Several underlying causes are conceivable due to complex interactions, such as perinatal stress and corresponding neurohumoral activation, maternal medication during anesthesia, and an increased risk of neonatal respiratory distress. Further analyses including these factors and differentiating between primary and secondary cesarean sections are needed to evaluate whether the birth mode indeed has an impact on the neonatal QT interval and what mechanisms might cause such an impact. To the best of our knowledge, no data have been published on this topic. As gestational age, birth weight, birth mode, and timing of the ECG were not the primary exposure variables in this analysis, these findings need to be verified in further studies.

Due to the heterogeneity of maternal drugs and the sample size of the respective medication groups, we performed a subgroup analysis for SSRIs, PPIs, and dimenhydrinate only. Although there are case reports of neonates with the prolonged QT interval associated with the maternal SSRI intake (Dubnov et al., 2005; Leerssen et al., 2019), two case–control studies performed on this topic had inflicting results. Although Dubnov-Raz et al. found a significant increase in the QT interval in intrauterine-exposed neonates, a recent study by Lindsay–Sutherland showed no differences in the two groups (Dubnov-Raz et al., 2008; Lindsay-Sutherland, 2021). The results in our cohort go in line with the latter study, showing no significant effect of SSRI exposure. No cases of neonatal QT interval prolongation after the maternal use of PPIs or dimenhydrinate have been described, and to the best of our knowledge, no studies addressing the effect of intrauterine exposure to these agents on the neonatal QT time have been conducted so far. In our analysis, there was no significant effect of intrauterine exposure on the neonatal QT interval in these two subgroups. Limiting in our setting, despite the considerably large cohort, the number of exposed infants in the respective subgroups was small.

This is, to the best of our knowledge, the first cohort study to investigate the possible effect of maternal medication with the known risk of QT interval prolongation in a multivariate analysis, taking important perinatal confounding factors into account. In summary, in our cohort, there was no significant effect on the neonatal QT interval, either in the overall or in the subgroup analyses, which adds important data on possible effects of maternal therapy with regard to this scarcely investigated neonatal adverse effect. Further studies with larger case numbers are necessary in order to have a more substantial database on the safety of these therapies and to clarify some of the contradictory previous study results.

In this analysis, the maternal medication use more than 4 weeks before delivery was defined as non-exposure (Med-). Although it is unlikely, it cannot be excluded that substances with a very long half-life still have residual activity and thus lead to fetal exposure at the time of birth. In addition, the prolonged use of long-acting drugs and subsequent accumulation can lead to increased fetal exposure.

As genetic variants are known to influence susceptibility for acquired QT interval prolongation (Kallergis et al., 2012), our results cannot be generalized to populations with different ethnic distributions. Furthermore, our results might be biased because of the single-center study design as maternal diseases and medication intake in our cohort may not match those of the general population. Future studies will need to take these factors into account when discussing individual aspects of medication safety.

## 5 Conclusion

A relevant number of women take potential QT-interval prolonging drugs during pregnancy and until delivery.

In our cohort, intrauterine exposure showed no effect on the neonatal QT interval and on the rate of infants with QT prolongation.

Further studies with larger case numbers are needed in order to have a more substantial database on the safety of these therapies.

Several perinatal confounders might have an influence on the neonatal QT interval. More data are needed to consider them appropriately in future studies or interpretation of neonatal ECGs in the clinical setting.

## Data Availability

The raw data supporting the conclusions of this article will be made available by the authors, without undue reservation.
